# An αA-crystallin gene mutation, Arg12Cys, causing inherited cataract-microcornea exhibits an altered heat-shock response

**Published:** 2009-06-04

**Authors:** Li-Yun Zhang, Gary Hin-Fai Yam, Pancy Oi-Sin Tam, Ricky Yiu-Kwong Lai, Dennis Shun-Chiu Lam, Chi-Pui Pang, Dorothy Shu-Ping Fan

**Affiliations:** Department of Ophthalmology and Visual Sciences, The Chinese University of Hong Kong, Hong Kong, China

## Abstract

**Purpose:**

To investigate the clinical features and molecular basis of inherited cataract-microcornea caused by an αA-crystallin gene (*CRYAA*) mutation in a Chinese family.

**Methods:**

A three-generation Chinese family with members having autosomal dominant cataract and microcornea was recruited. Genomic DNA from peripheral blood or buccal swab samples of five affected and five unaffected members were obtained. Based on 15 genes known to cause autosomal dominant cataract, single nucleotide polymorphisms (SNPs) or microsatellite markers were selected and genotyped for two-point linkage analysis. Direct sequencing was performed to identify the disease-causing mutation. The expression construct coding for recombinant COOH-terminal myc-His-tagged wild type or R12C αA-crystallin protein (CRYAA) was expressed in COS-7 cells. Detergent solubility and subcellular distribution of wild type and R12C CRYAA were examined by western blotting and immunofluorescence, respectively. Heat-shock response was monitored by quantitative polymerase chain reaction (qPCR) of heat-shock proteins 70 and 90α (HSP70 and HSP90α).

**Results:**

The five affected family members showed variable lens opacities and microcornea. Clinical features of cataract were asymmetric in two eyes of some affected subjects. A heterozygous missense substitution, c.34C>T, in *CRYAA*, which is responsible for the R12C amino acid change, segregated with autosomal dominant cataract (ADCC) in this family. This substitution was absent in 103 unrelated controls. When expressed in COS-7 cells, the R12C mutant CRYAA resembled the wild type protein in its solubility when extracted with 0.5% Triton X-100 and with its cytoplasmic localization. However, mutant cells exhibited an altered heat-shock response, evidenced by the delayed expression of HSP70, when compared to cells expressing wild type CRYAA.

**Conclusions:**

The R12C mutation in CRYAA was responsible for a variable type of inherited cataract associated with microcornea in this Chinese family. The altered heat-shock response of mutant cells suggested a change of chaperoning capacity and networking, which could be associated with the pathogenesis of hereditary cataract-microcornea syndrome.

## Introduction

Congenital or infantile cataract is an opacification of the lens, usually diagnosed at birth or shortly thereafter. Its prevalence is up to 6 in 10,000 live births, causing about 10% of childhood blindness worldwide [1-5]. This heterogeneous disease exhibits considerable phenotypic as well as genetic variation [6,7]. Genetically, autosomal dominant inheritance (ADCC) is the most common mode of transmission, though autosomal recessive and X-linked inherited forms have been reported. So far, over 26 genes with specific mutations have been identified to be associated with the onset or progression of hereditary cataract. Among them, 17 are found for isolated autosomal dominant cataract without other ocular or multisystem anomalies including genes coding for crystallins, membrane transport proteins, cytoskeletal proteins, transcription factors, and chromatin modifying proteins [8].

About 18% of inherited congenital cataracts appear with a distinct phenotype of congenital cataract-microcornea syndrome (CCMC; OMIM 116150) [9]. Six genes have been identified to associate with this particular defect including three lens-specific crystallin genes (namely crystallin αA (*CRYAA*) [10-13], crystallin βB1 (*CRYBB1*) [14], and crystallin γD (*CRYGD*) [10]) as well as genes encoding gap junction protein α8 (*GJA8*) [9,10], basic leucine zipper transcriptional factor (*MAF*), [15-17] and solute carrier, *SCL16A12* (a carboxylic acid transporter) [18]. Apart from that, one locus at 11q13 was reported to be related to microcornea and cataract formation, but no specific gene has yet been identified [19].

Here, we report a Chinese family exhibiting variable cataract phenotypes and variable microcornea/microphthalmia in an autosomal dominant transmission fashion. The R12C mutant CRYAA protein demonstrated an altered heat-shock response but no change of detergent solubility or subcellular redistribution. This phenotypic variation could represent a crucial role of CRYAA in cataract formation.

## Methods

### Subjects and clinical examinations

**Figure 1:**
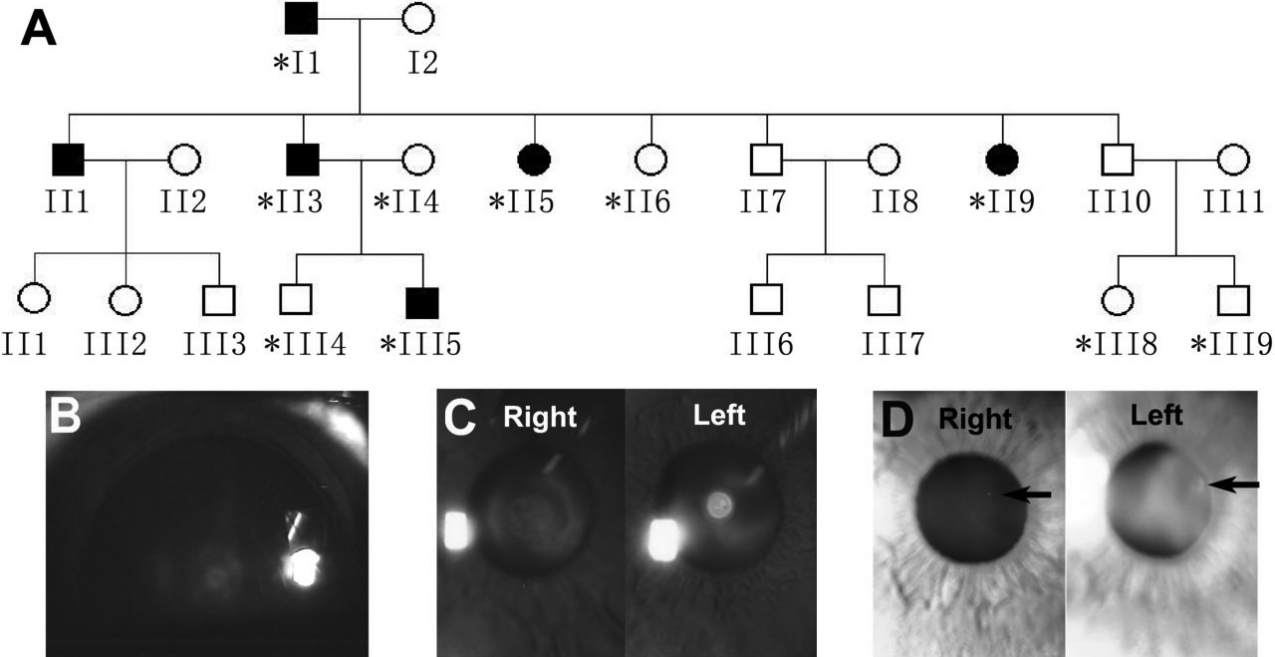
A Chinese autosomal dominant cataract family. **A**: Pedigree of a three-generation Chinese family with autosomal dominant cataract is shown. The asterisk indicates the family members whose DNA samples were analyzed. **B**: The lens image was taken from subject III5 following pupillary dilation. Both lenses showed the lamellar opacity within the fetal nucleus with a clear peripheral cortex. **C**: Subject II3 displayed similar lamellar opacity in the right lens and a central dense opacity with sharp margins in the left lens. **D**: Both images were from subject II5. A single spherical opacity (indicated by black arrows) was evident in the left lens, and only a smaller dot was found in the right lens.

A three-generation Chinese family with inherited cataract (Figure 1A) was recruited to the University Eye Center of The Chinese University of Hong Kong (Hong Kong, China). The research protocol followed the tenets of the Declaration of Helsinki and was approved by the ethics committee of The Chinese University of Hong Kong. Ten family members underwent complete ophthalmic examination including slit lamp biomicroscopy and ophthalmoscopy. All affected individuals (I1, II3, II5, II9, and III5) were followed up for eight years until 2008 by the same senior ophthalmologist in our eye center. During their last visit, additional ophthalmic examinations were performed including corneal diameter measurement, keratometry (Autokerato-refractometer KR-7100; Topcon Optical, Tokyo, Japan), anterior segment optical coherence tomography (1310 nm, Visante OCT Model 1000; Carl Zeiss Meditec, Dublin, CA), and ultrasonography (Ultrasonic Pachymeter SP-3000 and Biometer AL-100; Tomey Corporation, Nagoya, Japan). Slit lamp photography was also performed. Microcornea was defined as the horizontal corneal diameter being shorter than 11 mm [20,21] and microphthalmia as the antero-posterior axial length of the adult (normally elder than three) eye globe being shorter than 20 mm [22,23].

### Sample collection and DNA extraction

From five affected and five unaffected family members, whole blood samples from adult participants and buccal swab samples from children were collected. Genomic DNA was extracted using the QIAamp DNA kit (Qiagen, Valencia, CA). Unrelated subjects without cataracts were recruited as controls.

### Candidate gene screening

Fifteen known ADCC-associated genes were screened by linkage analysis. Thirty-nine single nucleotide polymorphism (SNP) markers based on 15 genes (Table 1) were selected with ABI SNP browser version 3.5 (Applied Biosystems, Foster City, CA). TaqMan SNP genotyping assay was performed by ABI PRISM 7000 sequence detection system (Applied Biosystems) followed by linkage analysis. For chromosomal regions in which SNPs were not informative, microsatellite marker analyses were used. Seventeen microsatellite markers flanking the target genes (Table 1) were chosen using the Marshfield genetic map. Genescan was performed with ABI PRISM^®^ 3130xl analyzer (Applied Biosystems). Two-point LOD scores were calculated by the MLINK subprogram from FASTLINK version 4.1P package. A gene frequency of 0.0001 and penetrance of 100% were assumed for ADCC.

**Table 1:** SNPs and microsatellite markers for candidate gene screening.

**Gene**	**Gene ID**	**Position**	**SNP**	**Microsatellite**
*CRYAA*	1409	21q22.3	rs870137	D21S1890
				D21S266
*CRYAB*	1410	11q22–22.3	rs2070894	
			rs1940392	
			rs14133	
			rs10502149	
*CRYBB1*	1414	22q11	rs4822752	
			rs5752351	
			rs2071859	
			rs4822749	
			rs5761618	
*CRYBB2*	1415	22q11.2	rs739315	
			rs5752084	
			rs969623	
*CRYBA1*	1411	17q11.1–12	rs8080081	D17S1873
*CRY GC*	1420	2q33–35	rs2242071	D2S2208
*CRY GD*	1421	2q33–35	rs2305429	
*GRY GS*	1427	3q25-qter	rs3774803	D3S1262
			rs11917060	D3S3570
			rs1447670	
			rs4686428	
*MIP*	4284	12q12	rs7953824	D12S1632
			rs2269348	D12S1691
			rs3809125	
			rs10876890	
*GJA8*	2703	1q21–25	rs7541950	
			rs2132397	
*GJA3*	2700	13q11–13	rs4769953	D13S1316
			rs1886176	D13S175
*BFSP2*	8419	3q21–22	rs1153876	D3S1290
			rs666067	D3S3713
			rs4854585	D3S3657
			rs6762405	D3S1292
			rs931099	
*PITX3*	5309	10q24–25	rs3758553	
*MAF*	4094	16q23.2	rs2288066	D16S3040
				D16S504
*HSF4*	3299	16q22	Hcv25749941	D16S3107
			Hcv25613880	
			rs11642409	
			rs9033	

This table showed all candidate genes studied in the screening experiment. The gene symbol, gene ID in NCBI, gene chromosome position, SNP markers and microsatellite markers for each gene were listed respectively.

### Gene sequencing

All coding exons and splice regions of *CRYAA*, *CRYBA1*, and *MAF* were sequenced with specific primers (Invitrogen, Carlsbad, CA; Table 2) using BigDye terminator v3.1 cycle sequencing kit (Applied Biosystems) on the ABI PRISM^TM^ 3130xl analyzer. Sequences were analyzed with reference to the NCBI GenBank (NM_000394 for *CRYAA*, NM_005208 for *CRYBA1*, and NM_001031804 and NM_005360 for *MAF*).

**Table 2:** Specific primers for *CRYAA*, *CRYBA1*, and *MAF* sequencing.

**Gene**	**Primer**	**Primer sequence (5′→3′)**
*CRYAA*	1F	CTCCAGGTCCCCGTGGTA
	1R	AGGAGAGGCCAGCACCAC
	2F	CTGTCTCTGCCAACCCCAG
	2R	CTGTCCCACCTCTCAGTGCC
	3F	AATGATCCTGCGATTTTGGAG
	3R	GGAAGCAAAGGAAGACAGACACC
*CRYBA1*	1F	CGCAGGGCTATAAAGAGGAG
	1R	GACAAGAGAAGGCTGTCTTCC
	2F	CCTTTCAAGGTATTCCCTCACC
	2R	CACTGGAGCTTGTGTGGACC
	3F	ATGTTCTAGCTCTCTTGCGC
	3R	GTAGTGATTTCTTTCGAGGCC
	4F	TGAACACCATGAACAAACACTAC
	4R	ACGGAAGTGGAAATTTCAGAG
	5F	CATGTGCTTCCTTGTATAATCC
	5R	ACTATTGATGCAACCTCAGG
	6F	CATCTCATACCATTGTGTTGAG
	6R	ACTTTCTAGAGTGCTTAGCAAGG
*MAF*	1F	CTCCTGCAGCCCATCTGG
	1R	CTGGTGGCTGTTGCTGATG
	2F	CATCAGCAACAGCCACCAG
	2R	GAGAAGCGGTCGTCGAAGT
	3F	ACTTCGACGACCGCTTCTC
	3R	TGGCGAGCATGGCTCTAG
	4F	CCTTTACGCTGCGTTTGATC
	4R	AACCCCCAGACAAGAGGC

F: forward primer; R: reverse primer.

### Expression constructs

A 548 base pair (bp) EcoR1/Xho1 fragment encompassing the human full length 519 bp open reading frame of wild type *CRYAA* was ligated to the EcoR1/Xho1 site of mammalian expression vector, pcDNA6/myc-His (Invitrogen). The c.34C>T mutation was introduced by polymerase chain reaction (PCR)-based site-directed mutagenesis (Stratagene, Lo Jolla, CA) using a specific oligonucleotide: sense 5′-ATC CAG CAC CCC TGG TTC AAG **T**GC ACC CTG GGG CCC TTC TAC C-3′ (the bold underlined alphabet indicated the specific base change). The constructs were verified by direct sequencing.

### Cell culture and transfection

COS-7 cells (ATCC, Manassas, VA) were maintained in Eagle’s Minimum Essential medium (Invitrogen) supplemented with 10% fetal bovine serum (FBS; Invitrogen) and 1% antibiotics at 37 °C in a humidified condition with 5% CO_2_ balanced with air. COS-7 cells were verified to contain negligible endogenous CRYAA by reverse transcription polymerase chain reaction (RT–PCR) and western blotting. CRYAA expression constructs were transfected to cells at a density of 1.5x10^4^ cells/cm^2^ by FuGene HD (Roche, Basel, Switzerland) at a ratio of 1 μg of DNA per 3 μl of FuGene HD in Opti-MEM® I supplemented with GlutaMAX^TM^ I (Invitrogen). Cells were collected for protein and mRNA analyses 24 h after transfection.

### Triton X-100 solubility assay

Cells with a density of 2.5×10^6^ cells/ml were lysed in a buffer containing 100 mM Tris-HCl (pH 7.4), 3 mM EGTA (Sigma, St Louis, MO), 5 mM MgCl_2_, 0.5% Triton X-100 (Tx; Sigma), protease inhibitor cocktail (Roche), and 1 mM phenylmethylsulfonyl fluoride for 2 min on ice. After centrifugation, the supernatant-containing Tx-soluble protein was collected and denatured in a sodium dodecyl sulfate (SDS) sample buffer containing 50 mM DTT. The pellet containing the Tx-insoluble protein was washed twice with ice-cold PBS, sonicated, and denatured in the SDS sample buffer containing 9 M urea. Tx-soluble and -insoluble proteins equivalent to 7.5×10^4^ cells were resolved by 15% SDS–polyacrylamide gel electrophoresis (PAGE) and by western blotting with mouse monoclonal antibodies against CRYAA (Abcam, Cambridge, UK), glyceraldehydes 3-phosphate dehydrogenase (GAPDH; Abcam), and β-actin (Sigma) and with rabbit polyclonal antibodies against myc (Santa Cruz, Santa Cruz, CA), His (Abcam), and appropriate horseradish peroxidase-conjugated Ig secondary antibodies (Jackson ImmunoResearch, West Grove, GA). The signals were detected by enhanced chemiluminescence (ECL; Amersham, Piscataway, NJ), and specific bands were quantified by Quantity One Image Analysis software (Bio-Rad, Hercules, CA). CRYAA expression was normalized with GAPDH (for Tx-soluble protein) or β-actin (for Tx-insoluble protein). Three independent experiments were performed, and paired Student’s *t*-test was used to determine the statistical significance of the data.

### Immunofluorescence

Cells were fixed with 2% paraformaldehyde (Sigma) in PBS (0.1 M, pH 7.4), permeabilized, and processed for immunofluorescence [24]. The sample was detected with mouse monoclonal anti-CRYAA antibody followed by rhodamine Red-X goat anti-mouse IgG secondary antibody (Invitrogen). After nuclear staining by 4'-6-diamidino-2-phenylindole (DAPI; Sigma), samples were examined with fluorescence microscopy (DMRB; Leica, Wetzlar, Germany) equipped with Spot RT color system (Diagnostic Instruments, Sterling Heights, MI).

### Heat-shock analysis

COS-7 cells expressing wild type or R12C CRYAA were heat-shocked at 42 °C for 20 min and then returned to 37 °C for different time intervals (0, 20, 40, 60, 120, 240, and 480 min). Total RNA was extracted with the RNeasy kit (Qiagen), quantified, and reverse-transcribed with a random primer (Roche) and SuperScript^TM^ III reverse transcriptase (Invitrogen). Real-time PCR was performed with SYBR Green/fluorescein PCR mix (Bio-Rad) and specific primers of *HSP70*, *HSP90α*, and *GAPDH* (Invitrogen; Table 3) using the ABI PRISM 7000 sequence detection system. Three independent experiments were performed.

**Table 3:** Expression primers of *HSP90A, HSP70*, and *GAPDH*.

**Gene**	**Primer**	**Primer sequence**
*HSP90A*	F	ACCCAGACCCAAGACCAACCG
	R	ATTTGAAATGAGCTCTCTCAG
*HSP70*	1F	AAGTACAAAGCGGAGGACG
	1R	GATGGGGTTACACACCTGC
	2F	TGCTGATCCAGGTGTACGAG
	2R	CGTTGGTGATGGTGATCTTG
*GAPDH*	F	GAAGGTGAAGGTCGGAGT
	R	GAAGATGGTGATGGGATTTC

F: forward primer; R: reverse primer.

### Computational analysis

Properties of the wild type and R12C mutant protein were analyzed by some software provided in the Expasy proteomics server. The isoelectric point (pI) and molecular weight (MW) were calculated by Compute pI/MW. The hydrophobicity change was predicted by ProtScale. The effect of the mutation was assessed by PolyPhen.

## Results

### Clinical features of family subjects

The pedigree exhibited an autosomal dominant pattern of transmission of infantile cataract (Figure 1A). Within the family, different phenotypes of lens opacity were observed in affected individuals between the two eyes of a subject. The proband (III5) was diagnosed with bilateral lamellar cataract at one year of age. The lamellar opacity was located in the fetal nucleus with a more dense center, and the peripheral lens was clear. During his last visit at the age of nine, his horizontal corneal diameters were 9.5 and 10 mm in the left and right eyes, respectively (Table 4). His father (II3) had different types of opacities between his eyes, which were first documented at 45 years of age, though he admitted to having eye problems in his younger age. The right lens had a lamellar type of opacification in the fetal nucleus whereas a dense nuclear cataract with a sharp margin was restricted in the left embryonic nucleus without affecting the fetal nucleus (Figure 1C). In his recent follow-up at the age of 53, the cataract showed no progression when compared to previous examinations, and the horizontal corneal diameters were 9.5 mm in both eyes (Table 4). Both II3 and III5 exhibited nystagmus, which might be related to the visual impairment during the early childhood [10]. The sister (II5) of II3 was first diagnosed with bilateral dot-like cataract in the cortical region at the age of 44 (Figure 1D). After eight years, the dotted opacity showed no progression, and the horizontal corneal diameter was approximately in the normal range (10.5 mm; Table 4). Another sister (II9) was identified to have variable cataract in both eyes at the age of 38. Dot-like opacity was present in the cortical region of one eye, and the lamellar type opacity was found in the other. The cataract had no obvious progression during the follow-up period. The horizontal corneal diameter was 10 mm when measured at age 46 years (Table 4). As a whole, no progression of lens opacity was noted in this family during their follow-up visits in the past eight years, and all eye examinations were performed by the same senior ophthalmologist in our eye center. Subject I1 was diagnosed to have age-related cataract with nuclear sclerosis on his first visit at 71 years of age. Since the lenses lost transparency, it was hard to determine whether lens opacification existed before or after nuclear sclerosis and the type of opacities. His horizontal corneal diameters were measured as 10 mm in both eyes at 79 years of age. All five affected members showed a microcornea phenotype with the horizontal corneal diameter (ranging from 9.5 mm to 10.5 mm) smaller than the defined 11 mm. Subjects II9 and III5 had microphthalmia. The antero-posterior length of the globe was less than 20 mm. All cataract subjects were phakic with mild to mediate vision impairment. Refractive errors included myopia, hyperopia, and astigmatism. The intraocular pressure of each subject was within normal range. They did not have other ocular abnormalities or systemic diseases.

**Table 4:** Clinical features of the affected family members.

**Patients**	**Age (years)**	**Gender**	**Eye**	**Best corrected visual acuity**	**Horizontal corneal diameter (mm)**	**Corneal curvature radius (mm)**	**Central corneal thickness (μm)**	**Average axial length (mm)**	**Type of cataract**	**Nystagmus**
I1	79	M	Right	20/50	10	6.72	550	20.11	Bilateral nucleus sclerosis	No
			Left	20/50	10	6.7	550	20.03		
II3	53	M	Right	20/35	9.5	6.86	520	21.45	Lamellar cataract	Yes
			Left	20/35	9.5	6.7	510	20.56	nuclear dense opacity	
III5	9	M	Right	20/40	10	6.91	550	18.85	Bilateral lamellar cataract	Yes
			Left	20/40	9.5	6.98	550	19.01		
II5	52	F	Right	20/35	10.5	6.68	570	21.83	Bilateral dot cataract	No
			Left	20/30	10.5	6.7	570	21.26		
II9	46	F	Right	20/30	10	6.88	530	19.73	Dot cataract	No
			Left	20/50	10	6.77	530	19.64	lamellar cataract	

The age given is when the ophthalmic examination was done. The average corneal diameter is 9.95±0.37 mm, the average corneal curvature radius is 6.79±0.11 mm, the average central corneal thickness is 543±20 µm, and the average axial length is 20.247±1.01 mm. M: Male; F: Female.

### Cataract-causing mutation

**Figure 2:**
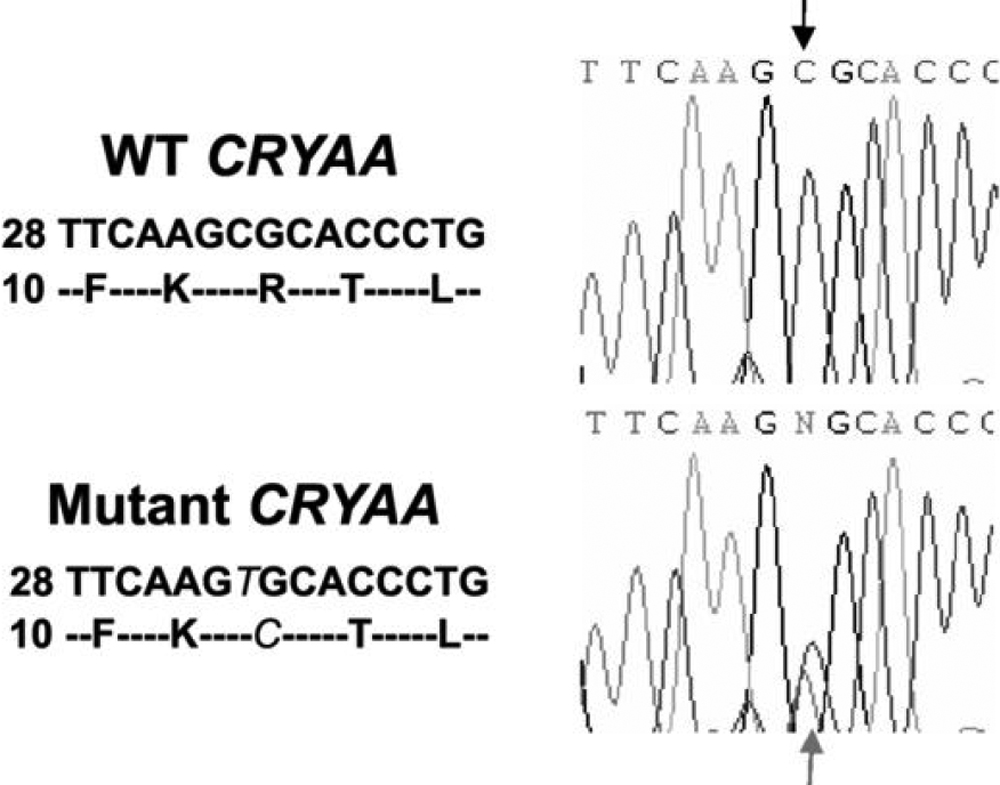
A novel R12C mutation of *CRYAA*. Sequencing results showed a substitution of C to T in *CRYAA* (c.34C>T), which led to a R12C amino acid change. In the upper panel, the wild type (WT) *CRYAA* DNA segment (starting from the coding position 28) and its coding amino acid sequence are listed on the left, and the sequencing image is shown on the right. The mutant DNA and polypeptide sequences and the sequencing image were shown in the lower panel. The mutation is indicated in italic. The sequence change in image is indicated by arrows.

Linkage analysis with SNPs and microsatellite markers was performed. Twelve of fifteen candidate cataract-causing genes were excluded. Three genes, namely *CRYAA* (D21S1890; LOD score 1.81), *CRYBA1* (D17S1873; LOD score 0), and *MAF* (D16S3040; LOD score 0), could not be screened because the selected markers were not sufficiently informative and were not directly sequenced. One heterozygous C>T transition at the 34th coding position (c.34C>T) in exon 1 of *CRYAA* (NM_000394) was identified (Figure 2). It caused a substitution of arginine with cysteine at the 12th amino acid position (R12C) and segregated with cataract in the family. Neither the unaffected members nor the 130 control subjects carried this base change. No sequence change was identified in *CRYBA1* or *MAF*.

### Protein feature of the R12C αA-crystallin mutation

**Figure 3:**
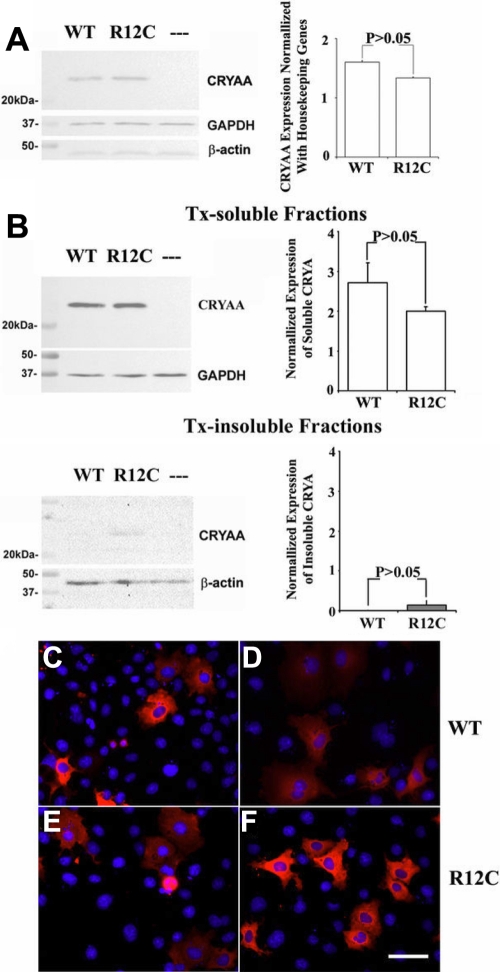
R12C CRYAA protein features. **A**: Western blot analysis is shown of total CRYAA expression in transfected COS-7 cells lysed with RIPA. The total amount of mutant R12C CRYAA expressed in COS-7 cells was similar to that of wild type (WT). “-” represents the CRYAA amount in cells transfected with vector only. The expression of housekeeping GAPDH and β-actin is shown as reference. Band densitometry verified that there was no significant difference between WT and R12C mutant amounts after being corrected by GAPDH expression. In band densitometry, bars represent SD. **B**: Detergent solubility of WT and R12C mutant is analyzed. The amount of R12C in Tx-soluble fractions was similar to that of WT. Only a trace of R12C mutant was detected in Tx-insoluble fractions. However, there was no significant difference between WT and R12C mutant amounts after normalization by the housekeeping protein expression. **C**-**F**: The subcellular distribution of WT and R12C CRYAA in COS-7 cells is analyzed as well. The transient expression of recombinant myc/His-tagged WT and R12C in COS-7 cells was detected by anti-CRYAA (**C** and **E**) and anti-myc antibodies (**D** and **F**), respectively. Both the WT (**C***,***D**) and mutant proteins (**E***,***F**) were predominantly localized in the cytoplasm. The red fluorescence indicates the expression of CRYAA, which was overlaid with DAPI-stained nucleus in blue color. Scale bar: 50 µm.

Recombinant myc/His-tagged wild type or R12C CRYAA was transiently expressed in COS-7 cells. In cell lysate extracted by radioimmunoprecipitation assay buffer, both the wild type and R12C CRYAA-myc-His fusion proteins migrated in a single band at approximately 25 kDa when examined by western blotting with monoclonal anti-CRYAA antibody or polyclonal anti-myc or anti-His antibody (Figure 3A). When transfected cells were extracted with 0.5% Tx and subsequently fractionated to obtain Tx-soluble and -insoluble proteins, the majority of R12C CRYAA was detectable in Tx-soluble fractions, similar to that of the wild type (Figure 3B). Only a trace of mutant protein was observable in Tx-insoluble fractions. After being normalized with GAPDH (for Tx-soluble proteins) or β-actin (for Tx-insoluble proteins), band densitometry revealed that the expression amount in either Tx-soluble or insoluble fractions had no significant difference between wild type and R12C CRYAA (p>0.05, Student’s *t*-test; Figure 3B). The results were consistent in triplicate experiments.

### Cytoplasmic distribution of wild type and the R12C αA-crystallin mutant

Immunofluorescence revealed that both wild type (Figure 3C,D) and R12C CRYAA (Figure 3E,F) were located mainly in the cytoplasm. Some transfected cells also showed minor nuclear staining of CRYAA, which might be due to the overexpression of the protein.

### Heat-shock responses

**Figure 4:**
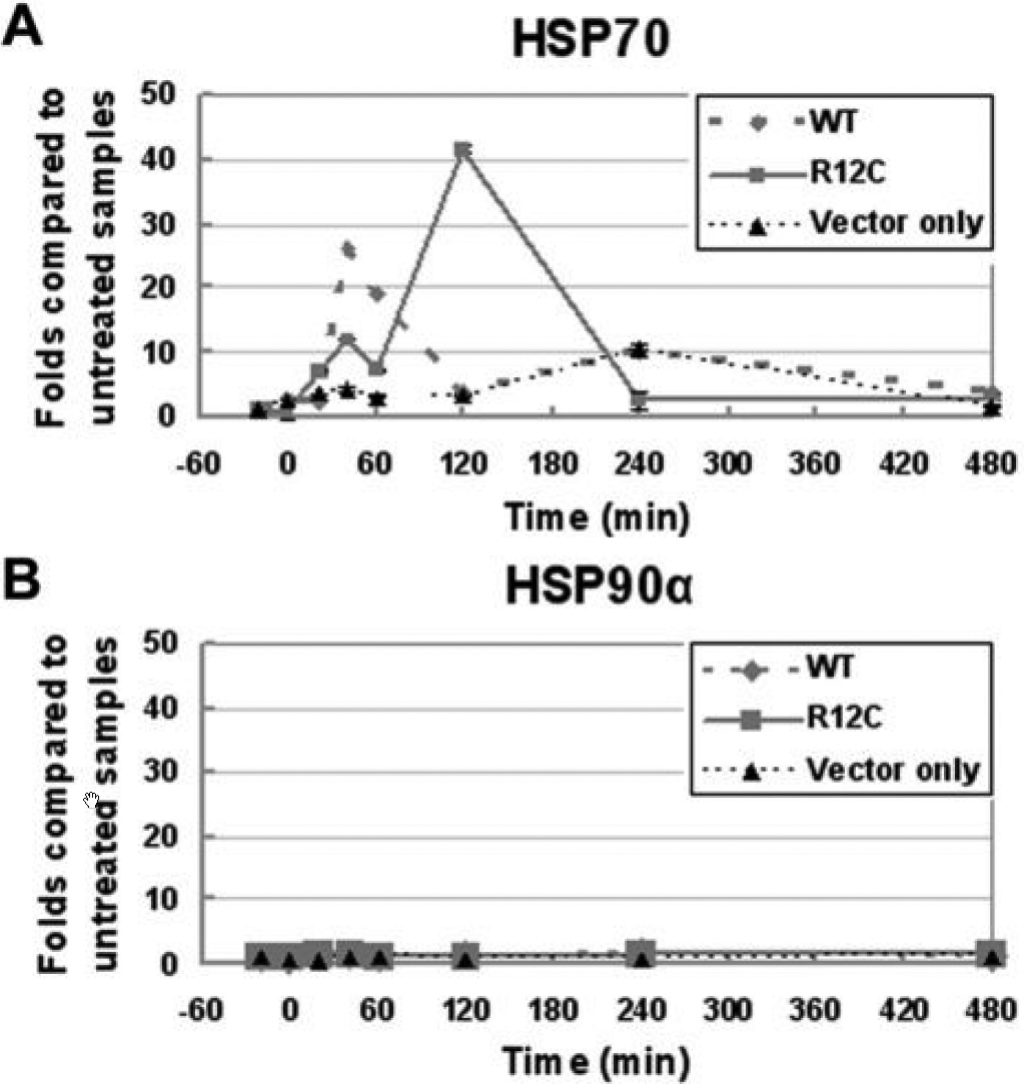
Time-dependent expression of *HSP70* and *HSP90α*. **A**: Time-dependent expression of *HSP70* is shown of cells expressing WT or R12C CRYAA and cells transfected with vector only. In WT CRYAA expressing cells, the *HSP70* expression reached to a peak level 40 min after heat shock while in R12C expressing cells, *HSP70* expression showed a biphasic curve and reached the highest level at 120 min. **B**: The *HSP90α* expression remained constant among all time points in cells expressing either WT or R12C CRYAA and in cells transfected with vector only.

COS-7 cells expressing wild type or R12C CRYAA were incubated at 42 °C for 20 min and then at 37 °C for different time intervals. Steady-state RNA expression of *HSP70* and *HSP90α* was investigated by quantitative PCR (qPCR). Each reaction was done in quadruplicate, and the mean signal reading was normalized with that of *GAPDH*. Data from different time points were compared to the non-heat-treated control. We detected a time-dependent change of the *HSP70* expression (Figure 4). For cells expressing wild type CRYAA, the *HSP70* RNA level began to increase shortly after cells were returned to 37 °C and reached a peak level after 40 min. The level was about 25 fold higher than the non-heat-treated control and about 12 fold higher than samples from cells transfected with the vector only. It was then reduced and maintained at a low level, close to that of the 0 min time. This pattern of changing *HSP70* RNA levels was shifted in cells expressing R12C CRYAA. A biphasic change was observed. The *HSP70* RNA level increased shortly after cells were returned to 37 °C incubation. The level was about 12 fold higher than that of the untreated control and about 6 fold higher than samples from cells transfected with vector only. The RNA level dropped to approximately 60% of the peak level followed by a second increase to a level about 40 fold more than that of the control and reached a peak at 120 min. It was then reduced and remained low for 480 min. Similar results were obtained in three independent experiments and when qPCR was performed using another pair of *HSP70* specific primers. For *HSP90α*, steady-state RNA levels remained constant in cells expressing either wild type or R12C CRYAA for all time points after heat stress (Figure 4).

### Computational analysis

**Figure 5:**
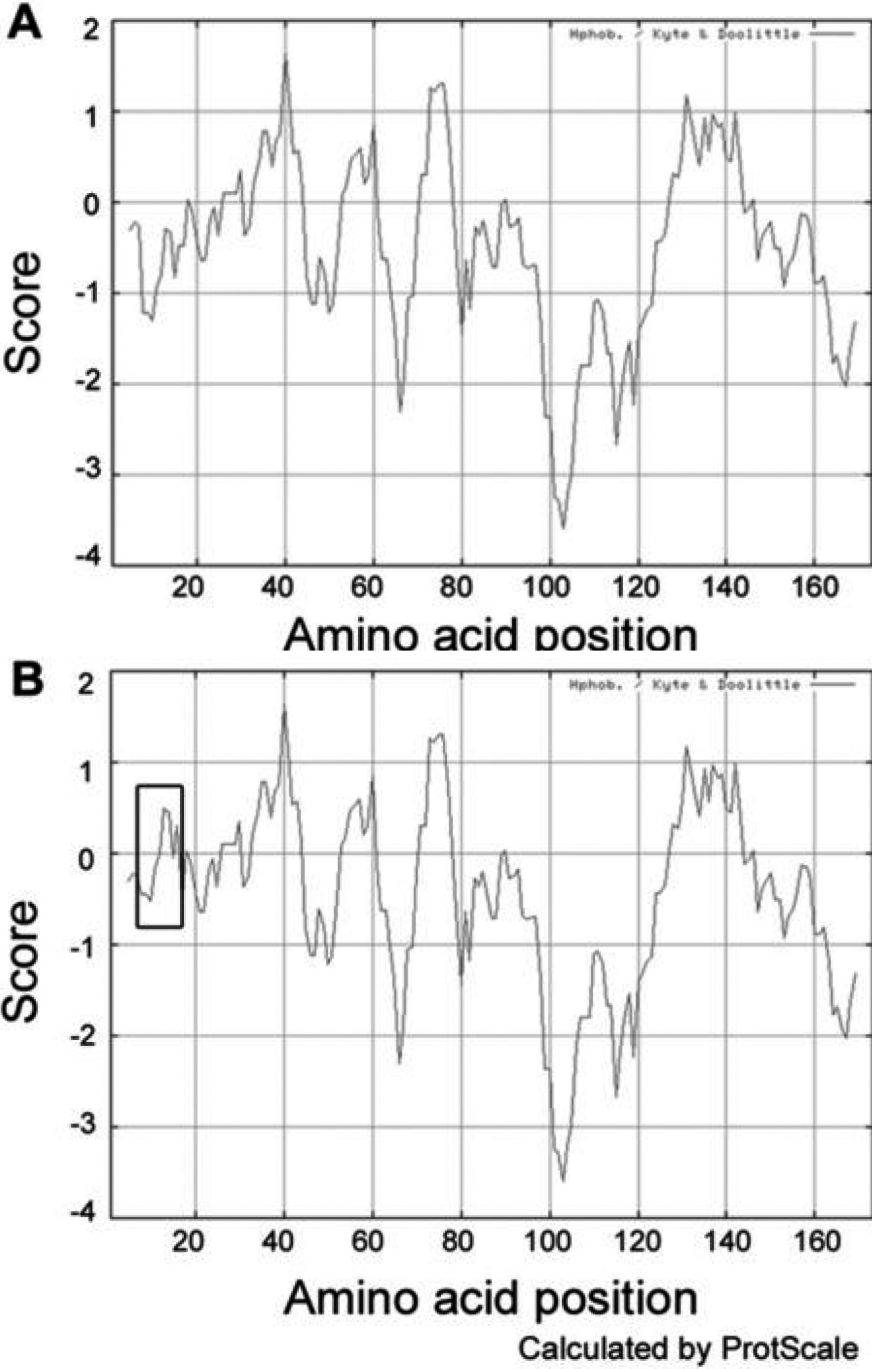
Altered hydrophobicity of R12C CRYAA protein. **A**: The hydrophobicity of WT CRYAA was predicted using the ProtScale program on the Expasy proteomics server. **B**: Compared to WT, R12C dramatically enhances the local hydrophobicity, which is indicated by the rectangle.

The theoretical pI of R12C CRYAA was slightly reduced to 5.6 compared to wild type CRYAA pI of 5.77. The MW of the mutant was reduced to 19,856 Da from the MW of wild type CRYAA of 19,909 Da. The R12C mutation caused no evident structural change to the protein by PolyPhen analysis. However, the ProtScale prediction illustrated an increase of local hydrophobicity around the substitution site (Figure 5).

## Discussion

In this study, we first reported a heterozygous mutation c.34C>T in exon 1 of *CRYAA* in a Chinese family affected with non-progressive variable inherited cataract and microcornea. This led to an amino acid substitution, R12C, close to the NH_2_-terminus of CRYAA.

Congenital cataract-microcornea (CCMC; OMIM 116150) syndrome is characterized by lens opacity and a corneal diameter smaller than 11 mm [20,21]. It can be associated with other ocular manifestations including myopia, iris coloboma, nystagmus, and microphthalmia [10,25]. One reported family with an autosomal recessive mode of inheritance had a mutation in *CRYAA* (R54C) [26]. Other CCMC (OMIM 116150) families inherited the disease in an autosomal dominant pattern [9-19]. Thirteen mutations in six genes have been identified to cause autosomal dominant CCMC (OMIM 116150). They include missense R12C, R21W, R116H, and R116C mutations of *CRYAA* [10-13]; a translational read-through mutation X253R of *CRYBB1* [14]; a premature truncation mutation, Y134X, of *CRYGD* [10]; V44E, P189L, and R198Q in *GJA8* [9,10]; R288P, K297R, and R299 in *MAF* [15-17]; and Q215X in *SCL16A12* [18]. Interfamilial, intrafamilial, and interocular variations of cataract phenotype could be caused by *CRYAA* mutations. For example, the R116C mutation in *CRYAA* led to fan-shaped cataracts in one family [12] and zonular central nuclear lens opacities in another family [13]. The affected members from a family carrying the R116H mutation of *CRYAA* exhibited variable lens phenotypes [11].

In this family, all cataract subjects had different severities of microcornea, some of them with microphthalmia. Although all affected subjects carried the R12C mutation, variable opacities among individuals and between eyes were presented within the family. The size and location of lens opacities had no obvious progression during the eight-year follow-up. The clinical features were compatible with the diagnosis of non-progressive cataract-microcornea with clinical variability. This phenotype was different from the Danish family described by Hansen’s [10] group with an affected individual carrying the R12C mutation showing posterior polar opacity progressing to dense nuclear and laminar cataract. The proband’s corneal diameter was 9.5 mm. Both the cataract progression and opacity morphology of this individual were different from our non-progressive cataract.

A total of nine *CRYAA* mutations have been reported to cause inherited cataract. Among them, R12C [10], R21W [10], R54C [26], R116C [12,13], and R116H [10,11] have been associated with cataract-microcornea syndrome. They are located in the highly conserved arginine residues in two major functional domains of the αA-crystallin, the crystallin domain near the NH_2_-terminus and the heat-shock domain near the COOH-terminus. The COOH-terminal domain was important for substrate-binding ability and chaperoning activity [27]. The NH_2_-terminus was reported to assist protein oligomerization, stability, and substrate binding [28]. It is reasonable to assume that changes in the NH_2_-terminus that disturb subunit binding would greatly affect the general functioning of the protein including reduced chaperoning activity. The substitution strongly underscores the importance of the bulky polar amino acid, arginine, in the conformation of the CRYAA protein and oligomeric assembly. Loss of arginine would cause a loss of positive charge and lead to a change of intra- or interpeptide bonding and folding. Functional studies have shown that loss of arginine destabilizes CRYAA, reducing its interactions with substrate proteins and chaperoning activity [29]. The phenotype of cataract is presumed to be caused by the reduced CRYAA molecular chaperoning ability on other lens proteins [30]. The associated microcornea might be due to the inductive effects from the abnormally formed lens on the cornea during embryogenesis [13]. There are other mutations in *CRYAA* reported to cause a cataract phenotype without microcornea. R21L [31], R49C [32], and G98R [33] were associated with the autosomal dominant form while W9X [34] was associated with the autosomal recessive mode. This divergence of *CRYAA* mutations causing cataract-microcornea syndrome or cataract alone could have different explanations. It is probable that some lens changes may affect cornea development while others do not. Moreover, the corneal size changes might not be obvious in some cases and thus are neglected in routine examinations.

To demonstrate the effect of R12C substitution on CRYAA chaperoning activity, we used a cell heat stress model. Myc-tagged recombinant CRYAA was expressed in COS-7 cells, which were subjected to heat stress. The heat-shock response was monitored by the expression of heat-shock proteins. We demonstrated that *HSP70* expression was different in cells expressing R12C CRYAA whan compared to wild type CRYAA. Stress-inducible HSP70 is one chaperone molecule that cooperates with multiple other chaperones [35] to assist in folding and to reduce aggregation of client proteins [36,37] and permanently disposes misfolded proteins via the lysosomal [38] or ubiquitin-proteasome pathway [39]. It was noted that *Hsp70* was strongly and specifically expressed in the embryonic lens of zebrafish for a short period of time and that it was required for normal lens formation [40,41]. Similar expression was also detected in embryonic chicken and human lens [42-44]. In zebrafish, the inhibition of *Hsp70* expression may lead to small eye phenotype, characterized by underdeveloped lens [45]. In our study, R12C CRYAA expressing cells exhibited an altered *HSP70* expression when compared with cells expressing wild type CRYAA. The result suggested that the R12C mutation causes CCMC in our Chinese family possibly through delaying or decreasing *HSP70* expression during lens development.

Protein analysis by ProtScale clearly showed an increase of local hydrophobicity around the Arg-Cys substitution site in CRYAA (Figure 5). As exhibited in other lens proteins, hydrophobicity is associated with crystallin activities. Increased hydrophobic interaction could reduce solubility of CRYBB2 [46]. Normal folding of CRYGD was maintained by the hydrophobic interface [47,48]. For CRYAA, hydrophobicity was involved in protein oligomerization and chaperoning activity [49,50]. Hence, the predicted shift of hydrophobicity due to the R12C change might alter the local protein structure and contact interfaces, which subsequently results in changes of protein function. Using PolyPhen analysis to predict the effect of amino acid change on protein structure and function, R12C CRYAA did not cause major structural changes when compared to the wild type. Additionally, a slight reduction of theoretical isoelectric point from 5.77 to 5.60 and of molecular weight from 19,909 to 19,856 Da was noted. Their effect on the CRYAA structure has to be further investigated.

In conclusion, the c.34C>T mutation in *CRYAA* that leads to the R12C substitution segregated with stable inherited cataract in a Chinese family. It was associated with microcornea and microphthalmia. Expression of R12C CRYAA with altered chaperoning capability modified the heat-shock response of affected cells. Such an altered CRYAA property may be related to the development of CCMC (OMIM 116150).
